# Impact of evergreening on patients and health insurance: a meta analysis and reimbursement cost analysis of citalopram/escitalopram antidepressants

**DOI:** 10.1186/1741-7015-10-142

**Published:** 2012-11-20

**Authors:** Ali A Alkhafaji, Ludovic Trinquart, Gabriel Baron, Moïse Desvarieux, Philippe Ravaud

**Affiliations:** 1 Centre d'Epidémiologie Clinique, Hôpital Hôtel-Dieu, Assistance Publique-Hôpitaux de Paris, 1 place du parvis Notre Dame, Paris, 75004, France; 2 INSERM U738, Hôpital Hôtel-Dieu, Assistance Publique-Hôpitaux de Paris, 1 place du parvis Notre Dame, Paris, 75004, France; 3 Université Paris Descartes - Sorbonne Paris Cité, 12 Rue de l'École de Médecine Paris, 75006, France; 4 French Cochrane Centre, Hôpital Hôtel-Dieu, 1 place du parvis Notre Dame, Paris, 75004, France; 5 EHESP School of Public Health, Avenue du Professeur Léon-Bernard, CS 74312, Rennes, 35043, France; 6 Department of Epidemiology, Columbia University Mailman School of Public Health, 722 West 168th Street, New York, NY 10032, USA

**Keywords:** Evergreening, Meta-analysis, Health Insurance Reimbursement, Escitalopram, Citalopram, Chiral switch, French health information system, generic drugs

## Abstract

****Background**:**

"Evergreening" refers to the numerous strategies whereby owners of pharmaceutical products use patent laws and minor drug modifications to extend their monopoly privileges on the drug. We aimed to evaluate the impact of evergreening through the case study of the antidepressant citalopram and its chiral switch form escitalopram by evaluating treatment efficacy and acceptability for patients, as well as health insurance costs for society.

****Methods**:**

To assess efficacy and acceptability, we performed meta-analyses for efficacy and acceptability. We compared direct evidence (meta-analysis of results of head-to-head trials) and indirect evidence (adjusted indirect comparison of results of placebo-controlled trials). To assess health insurance costs, we analyzed individual reimbursement data from a representative sample of the French National Health Insurance Inter-regime Information System (SNIIR-AM) from 2003 to 2010, which allowed for projecting these results to the whole SNIIR-AM population (53 million people).

****Results**:**

In the meta-analysis of seven head-to-head trials (2,174 patients), efficacy was significantly better for escitalopram than citalopram (combined odds ratio (OR) 1.60 (95% confidence interval 1.05 to 2.46)). However, for the adjusted indirect comparison of 10 citalopram and 12 escitalopram placebo-controlled trials, 2,984 and 3,777 patients respectively, efficacy was similar for the two drug forms (combined indirect OR 1.03 (0.82 to 1.30)). Because of the discrepancy, we could not combine direct and indirect data (test of inconsistency, *P *= 0.07). A similar discrepancy was found for treatment acceptability. The overall reimbursement cost burden for the citalopram, escitalopram and its generic forms was 120.6 million Euros in 2010, with 96.8 million Euros for escitalopram.

****Conclusions**:**

The clinical benefit of escitalopram versus citalopram remains uncertain. In our case of evergreening, escitalopram represented a substantially high proportion of the overall reimbursement cost burden as compared with citalopram and the generic forms.

## Introduction

Evergreening refers to owners of pharmaceutical products using numerous strategies, such as patent laws and minor drug modifications, to extend their monopoly privileges with their products [1-3]. Typically, these strategies are developed before expiry of the patent of an original drug, usually a high-revenue drug [4-6]. If they succeed, they result in an extension of the patent protection period or a new patent for a minimally modified version of the drug.

A consequence of evergreening is delayed entry of generic drugs into the market with extension of the original drug patent or competition between the patent-protected minimally modified version of the drug and generic drugs [7]. This situation might increase drug reimbursement costs by keeping the cheaper generic versions completely or partly out of the market [8]. Pharmaceutical companies defend evergreening practices and claim that revised formulas benefit patients (for example, by improving adherence) and the drug industry (for example, by providing incentives for companies to engage in incremental innovation) [9-11].

Minimal modifications used in evergreening include use of a different salt or molecule as an additive to the main drug components, change in formulation, modified release or change in route of administration [12,13]. An enantiomer patent is another form of evergreening based on a chiral switch (that is, from a chiral drug developed as a racemic mixture to a single enantiomer) [14]. Single-enantiomer drugs represent more than 50% of the top-selling 100 drugs worldwide [15].

A typical example of this strategy was the case of citalopram/escitalopram, two antidepressants. The Lundbeck company's patent on citalopram has run out in many countries [15]. The company launched a single-enantiomer drug, escitalopram, before the patent on the original drug expired and significantly increased its advertising campaigns to promote the new form [16]. However, the clinical superiority of escitalopram over citalopram is still debated [15]. Moreover, previous evergreening's societal burden analyses, dedicated to other evergreening examples, were based on projections of market shares and health insurance reimbursement costs [4,17].

We aimed to evaluate the impact of evergreening citalopram with the chiral switch form escitalopram on efficacy and treatment acceptability for patients, as well as health insurance costs for society.

## Methods

We investigated the relative efficacy and acceptability of citalopram and escitalopram by performing meta-analyses for direct evidence (meta-analyses of results of head-to-head trials) and indirect evidence (adjusted indirect comparison of results of placebo-controlled trials). Health insurance costs were analyzed through reimbursement data for citalopram, its generic forms and escitalopram from the French national health insurance information system.

### Assessment of relative efficacy of escitalopram and citalopram

#### Identification and selection of randomized controlled trials

We identified randomized controlled trials by systematically identifying reviews published from 2000 to 2011 and trial results published from 2011 to 2012 [18], see Section 1, Additional file 1.

Eligible reviews assessed the efficacy of citalopram or escitalopram in adults with major depression based on randomized trials. We searched several bibliographical databases for reviews published between January 2000 and March 2011, and four repositories of national health technology agencies, as well as the FDA. See Section 1, Additional file 1.

Eligible randomized trials assessed acute treatment efficacy, which was defined as eight-week treatment efficacy of citalopram versus escitalopram or citalopram and/or escitalopram versus placebo in patients with major depression, see Section 1, Additional file 1 for detailed selection criteria. First, we screened selected reviews and listed all included trials. The eligibility of trials was assessed independently by two reviewers, with disagreements resolved by consensus. Then, we searched for trial results published from March 2011 to February 2012 in MEDLINE and EMBASE. Finally, we searched for trial results in databases from Lundbeck and Forest registries [19,20]. We also contacted Lundbeck/France for a list of clinical trials for the two medications.

#### Outcome measures

We assessed acute treatment efficacy, which was defined as eight-week treatment. When the depression outcome was measured at several timepoints, we extracted outcome data at eight weeks. If not reported, we extracted outcome data for the closest time points, ranging from 4 to 12 weeks [21,22], see Sections 1 and 2, Additional file 1 for details about data extraction. We used outcome data for the Montgomery-Åsberg depression rating scale (MADRS) and, if not reported, the Hamilton scale. Efficacy was assessed by the proportion of responders in each treatment group, defined as patients with a decrease in depression score from baseline to follow-up of at least 50%, see Section 2, Additional file 1.

We assessed treatment acceptability by the proportion of patients who did not drop out of the allocated treatment during the short-term treatment period (completers), see Section 2, Additional file 1. Data were extracted by two reviewers independently. Disagreements were resolved by discussion. If outcome data were available from FDA reports and other sources, priority was given to FDA data, because the FDA re-analyses of raw data from the sponsor adhered to the pre-specified statistical methods in the trial protocol [23].

#### Meta-analysis of head-to-head trials and adjusted indirect comparisons of placebo-controlled trials

First, we performed a meta-analysis of head-to-head trials. Then, we performed an adjusted indirect evaluation of the relative efficacy of each treatment compared to placebo using Bucher's method [24]. The effect of treatment was measured by odds ratios (ORs) and 95% confidence intervals (95% CIs). Combined estimates were calculated by fixed- and random-effects models. The two models always showed similar results [25]. In cases of significant treatment effect, we re-expressed the results in terms of number needed to treat (NNT). We computed the NNT from the combined ORs and by considering low and high response rates for the control group, defined as the lower and upper bounds of the 95% CI for the combined response rate across control groups in the meta-analysis.

We assessed heterogeneity of treatment effect estimates across trials using the I² statistic. We assessed similarity (whether citalopram and escitalopram placebo-controlled trials were similar for moderators of relative treatment effect) by comparing clinical and methodological characteristics of randomized comparisons [26]. We assessed inconsistency between the direct and adjusted indirect estimate by the difference between the two estimates and associated 95% CIs and tested whether it was statistically significant [27].

We assessed small-study effects by funnel plots [28]. Sensitivity analyses for direct and adjusted indirect comparisons involved re-analyzing data after excluding head-to-head trials without comparable dosages and excluding placebo-controlled trials as soon as the treated group did not receive defined daily dose (DDD) dosages, respectively. In addition, sensitivity analyses were performed excluding trials with imputed outcome data and trials of older adults only.

### Assessment of reimbursement costs for citalopram, its generic drugs and escitalopram

To assess the reimbursement burden of the three drug forms on the French general health insurance regimes, we analyzed data from the French national health insurance information system (Système National d'Information Inter-Régimes de l'Assurance Maladie, SNIIR-AM).

#### Data sources

The SNIIR-AM contains anonymous and comprehensive data on health spending reimbursements from 2003. In 2009, it covered 86% of the French population, approximately 53 million people. We used a random representative sample (1/97) of SNIIR-AM, the Echantillon généraliste de bénéficiaires (EGB). In 2009, the EGB consisted of about 500,000 beneficiaries [29,30]. We searched the EGB database using French identifiers for drug products for the three drug forms. We examined all reimbursement claims for the drugs between January 2003 and December 2010, given that generic citalopram was introduced in December 2003 and escitalopram was introduced in June 2005 in France.

#### Reimbursement cost analysis

To illustrate changes in consumption pattern, we calculated the monthly number of reimbursements and monthly consumption in DDD units for the three drug forms. According to the World Health Organization, the DDD is the assumed average maintenance dose per day for a drug used for its main indication in adults. The DDD is 20 mg for citalopram and 10 mg for escitalopram [31]. To illustrate the evolution of spending, we calculated the total monthly reimbursement costs for the three drug forms. For all data, we produced time series plots. Because the EGB sample is representative of the SNIIR-AM population, we projected our analyses by dividing all results by the sampling fraction so that estimates reflected the whole SNIIR-AM population [30].

Analyses involved use of Stata MP v10.0 (Stata Corp., College Station, TX, USA). A *P *< 0.05 was considered statistically significant.

## Results

### Assessment of relative efficacy of citalopram and escitalopram

**Figure 1:**
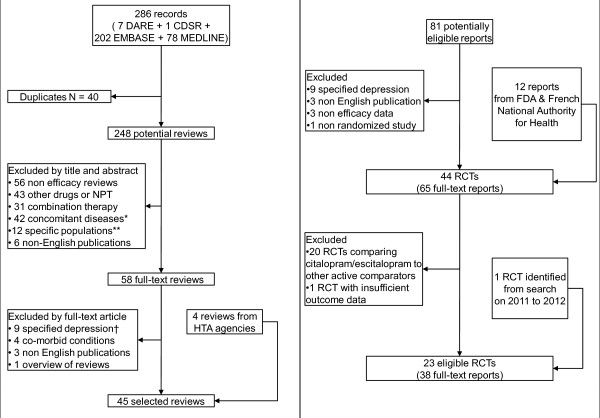
**Flow diagram of systematic reviews selection (left panel) and eligible randomized trials (right panel)**. *Concomitant diseases: depression in patients with, for example, fibromyalgia and diabetes. **Specific populations: children, pregnant women. †Specified depression: end-of-life depression, postpartum depression.

Of 248 records, we selected 41 reviews (Figure 1). In addition, we identified four reports from health technology assessment agencies. From the 45 selected reviews, we identified 81 full-text reports concerning potentially eligible randomized clinical trials and we selected 22 eligible randomized controlled trials (RCTs) (Figure 1). Search for trials, from 2011 to 2012, yielded one additional eligible trial [32]. No additional trial from manufacturer registries or Lundbeck's list of trials was identified.

For the 23 RCTs, 4 had published results only, 6 had unpublished results only and 13 had both published and unpublished results. The trials provided for 29 randomized comparisons: 7 between citalopram and escitalopram, 12 between escitalopram and placebo and 10 between citalopram and placebo; 3 trials provided a closed loop comparison of citalopram, escitalopram and placebo (See Sections 3 and 4, Additional file 1).

A total of 2,569, 2,412 and 2,376 participants were allocated to escitalopram, citalopram and placebo, respectively. Elderly patients were included in four trials. Outcome assessment times ranged from 4 to 12 weeks. All trials were sponsored by pharmaceutical companies, except one by the Chinese National Institute for Pharmaceutical Research, (See Section 5, Additional file 1).

#### Meta-analysis of head-to-head trials

Of seven identified head-to-head randomized comparisons, all showed the superiority of escitalopram over citalopram except Ou 2011 [32]. Escitalopram was associated with higher response as compared with citalopram (random-effects model, combined OR 1.60 (95% CI 1.05 to .46)) (See Section 8, Additional file 1). This combined OR would translate to a NNT of 8.5 and 9.6 patients to achieve an additional response with escitalopram compared to citalopram, when the control response rate is lower (47%) or higher (61%). Heterogeneity was considerable across trials (I² = 80%; τ² = 0.26), but mainly because of one trial, Yevtushenko 2007, which showed outlying results. The funnel plot of the seven comparisons did not reveal asymmetry; see Section 9, Additional file 1.

Concerning acceptability, the proportion of treatment completers was greater with escitalopram than citalopram (Section 10, Additional file 1). For escitalopram versus citalopram, the random-effects combined OR was 1.27 (0.93 to 1.72), with moderate heterogeneity (I² = 26% and τ² = 0.04).

#### Adjusted indirect comparisons of placebo-controlled trials

For the two meta-analyses of placebo-controlled comparisons of citalopram (n = 10 trials) and escitalopram (n = 12), we found no substantial heterogeneity across trials (I² = 0% and τ² = 0.00 for citalopram vs. placebo; I² = 27% and τ² = 0.02 for escitalopram vs. placebo), Section 6, Additional file 1. Patients and trial characteristics were similar for the two sets of placebo-controlled trials, see Section 6, Additional file 1.

**Figure 2:**
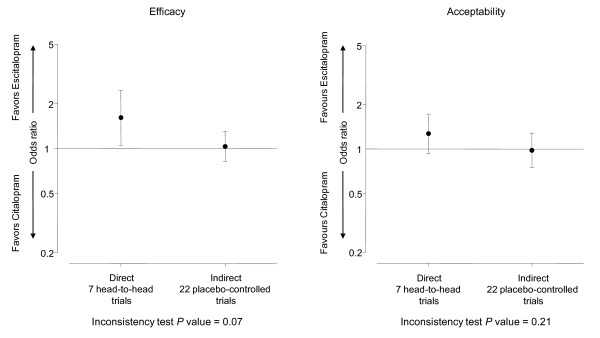
**Direct and indirect comparisons of escitalopram and citalopram for efficacy and acceptability**.

The proportion of responders was significantly greater with citalopram and escitalopram than placebo and the two effect sizes were of similar magnitude. Random-effects combined OR 1.50 (1.27 to 1.78) for citalopram and 1.55 (1.33 to 1.82) for escitalopram. From these estimates, the adjusted indirect comparison OR for citalopram versus escitalopram was 1.03 (0.82 to 1.30). We found a large inconsistency between the direct and indirect estimates (difference in log ORs 0.44, corresponding to a ratio of OR of 1.55, *P *= 0.07, Figure 2. Consequently, we could not combine the direct and indirect estimates in a network meta-analysis. Moreover, we could not perform a NNT analysis because of a lack of difference in efficacy between citalopram and escitalopram.

For each set of placebo-controlled trials, we found no evidence of small-study effect (See Section 12, Additional file 1; Egger's test *P *= 0.79 for citalopram vs. placebo and *P *= 0.46 for escitalopram vs. placebo).

Concerning acceptability, the proportion of treatment completers was lower with citalopram and escitalopram than placebo, with similar effect sizes, see Section 13, Additional file 1, the random-effects combined OR 0.91 (0.75 to 1.10) for citalopram and 0.89 (0.73 to 1.07) for escitalopram. From these estimates, the adjusted indirect comparison OR for escitalopram versus citalopram was 0.98 (0.75 to 1.28), with large inconsistency between the direct and indirect estimates (difference in log ORs 0.26, corresponding to a ratio of OR of 1.30 (*P *= 0.21), Figure 2. We found no important heterogeneity in the two sets of trials (I² = 0% and τ² = 0.00 for citalopram vs. placebo; I² = 25% and τ² = 0.03 for escitalopram vs. placebo).

The sensitivity analyses of the direct and indirect comparisons after excluding trials without comparable dosages, with imputed outcome data or of older adults only gave results consistent with those from the primary analyses (data not shown).

### Assessment of reimbursement costs for citalopram, its generic drugs and escitalopram

Sections 7 and 14, Additional file 1 show the evolution of the number of claims for each drug form. The projected results from the EGB sample showed a substantial decrease in consumption of citalopram between 2004 (2.1 million claims) and 2006 (0.7 million claims). This decrease was accompanied by an increase of approximately twice the claims for the generic forms of citalopram during the same period. Moreover, the new revised-formula escitalopram represented 40% of the market share in 2006 (1.7 million claims) after it was introduced to the market in April 2005. Between 2006 and 2011, claims for citalopram continued to decrease, to a lesser extent, and that for the generic forms continued to increase, to peak in 2008, which was followed by a slight decrease up to 2010. However, escitalopram claims grew even more steeply towards the end of 2010, reaching 5.4 million claims. By the end of 2010, escitalopram consumption had exceeded that of citalopram and its generic forms combined (5.4 million claims for escitalopram vs. 0.2 and 1.7 million for citalopram and its generic forms, respectively) (Sections 7, 16 and 17, Additional file 1).

Consumption in DDD units showed changes similar to reimbursement results; for citalopram consumption, the DDD units decreased from 73.9 million in 2004 to 7.6 million in 2010, whereas for escitalopram, the units increased from 15.7 million in 2005 to 193.9 million in 2010. For generic forms of citalopram, the DDD units were 55.2 million in 2005 and slightly increased to 58.8 million in 2010 (Sections 7 and 15, Additional file 1).

**Figure 3:**
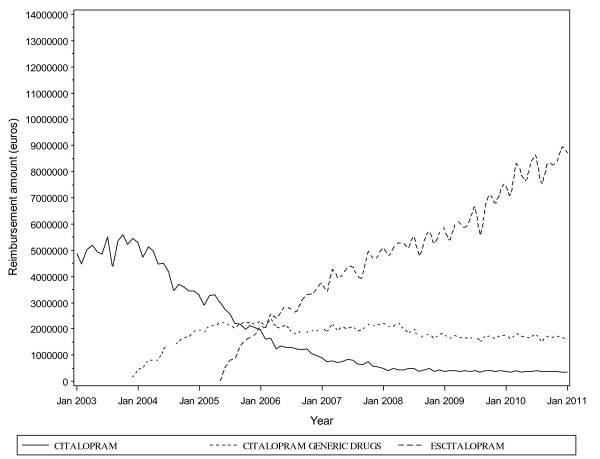
**Reimbursement costs (monthly reimbursement expenditure in Euros) between 2003 and 2011 in France**.

**Figure 4:**
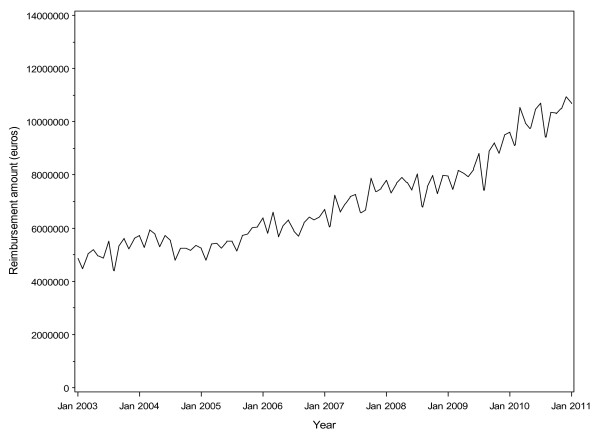
**Total monthly reimbursement cost for escitalopram, citalopram and its generic forms**.

**Figure 5:**
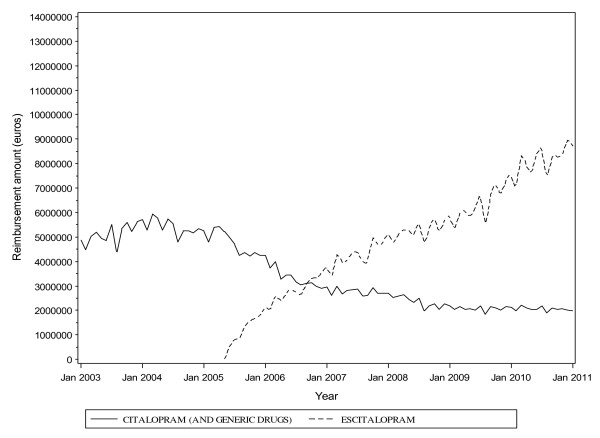
**Total monthly reimbursement cost for citalopram and its generic forms combined versus escitalopram**.

Reimbursement costs reflected the trends in consumption (Figure 3; Section 7, Additional file 1). The total monthly cost for the drugs was 5.6 million Euros when the generic forms were introduced into the market. Although the total monthly cost slightly decreased to 5.2 million Euros when escitalopram was introduced into the French market, in May 2005, the monthly cost reached 6.1 million Euros a year subsequently (Figure 4). The cost burden of escitalopram continued to increase, to reach 96.8 million Euros in 2010, as compared with citalopram, 4.4 million Euros (Figure 3). For the generic forms of citalopram, the cost was >20 million Euros from 2005 to 2010. Moreover, the reimbursement cost for escitalopram exceeded that of citalopram and its generic forms combined (Figure 5). Overall, the health cost burden of the three drug forms reached 120.6 million Euros in 2010 (see Figure 5 and Section 17, Additional file 1).

## Discussion

We performed a large-scale systematic review to evaluate the impact of evergreening of citalopram with the chiral switch form escitalopram on efficacy, treatment acceptability and health insurance costs. We found substantial discrepancy between the direct and indirect comparisons of citalopram and escitalopram for short-term treatment efficacy and acceptability. The direct comparison showed clinical superiority of escitalopram over citalopram, but the indirect comparison showed no evidence of difference between the two. Our analysis of reimbursement costs for a large representative sample of French health insurance beneficiaries, showed that escitalopram took a large share of the market, with citalopram substantially lower. The generic forms of citalopram started to gain an expected share of the market, but the uptake was suppressed by escitalopram competition. Moreover, escitalopram consumption overcame citalopram and its generic forms combined.

Network meta-analysis can be used to combine direct and indirect estimates of efficacy or acceptability. The analysis borrows strength from all the available evidence and increases statistical power and precision of estimates. However, we could not perform such a network meta-analysis because of the observed discrepancy between direct and indirect evidence. A possible explanation for the discrepancy is dose difference among trials; however, sensitivity analysis for comparable dosages showed consistent results. Another possible explanation could be bias in the direct comparison or the indirect comparison [27,31]. Head-to-head trials are usually considered the gold standard. However, such trials may favor the sponsored treatment [33,34] or the newest treatment [35-37]. In our analysis, most head-to-head trials, except two, were sponsored by the manufacturer (Lundbeck/Forest Lab). One of the two trials was sponsored by Arbacom, a Russian company with potential conflict of interest with the Danish manufacturer Lundbeck [38]. The trial results contained outlying results, with strong superiority of escitalopram over citalopram. The second trial was an institutional funded trial [32], and did not find any evidence of difference between the two drugs. Escitalopram was superior to citalopram in a network multiple-drug treatments meta-analysis of efficacy without placebo controlled trials [22]. However, citalopram was superior to escitalopram, with a statistical non-significance, in an extensive multiple-drug treatments meta-analysis that considered the entire network of second-generation antidepressant drugs and including placebo-controlled trials [21].

Our indirect comparison may have been biased. For instance, the characteristics of citalopram and escitalopram placebo-controlled trials may have greatly differed, which would have invalidated the adjusted indirect comparison. However, we found no evidence of unequal distribution of potential treatment effect modifiers. Placebo-controlled trials may have exhibited reporting bias. Nevertheless, this bias is unlikely because as compared with active comparator trials, placebo-controlled trials are frequently registered with the FDA, which is considered a gold standard for placebo-controlled trials in the antidepressant field [39] and when we searched the FDA database, we identified five trials with unpublished data. As well, the indirect comparison may have had low statistical power [40]. However, we ensured a balance in the number of included trials, with at least 10 randomized comparisons for both citalopram and escitalopram versus placebo.

Our study has some limitations. In the comparative effectiveness analysis, we assessed treatment acceptability only and did not assess safety outcomes [21,22]. Our findings could not be generalized to other patent-extended medications using chiral switch or other indications for citalopram/escitalopram usage. Second, we examined the EGB sample [29], although the EGB is a representative sample of the SNIIR-AM database [30]. The EGB data limited our analysis to begin with 2003, so we were not able to look at the cost and consumption trends earlier than 2003.

## Conclusions

We found strong uncertainty about the clinical benefits of escitalopram over citalopram. Given the likelihood of sponsorship bias for head-to-head trials and the absence of reporting bias for placebo-controlled trials [41], our adjusted indirect comparison may be less biased than the direct comparison. However, the market share of escitalopram increased substantially and suppressed that of the generic forms, which may have prevented a substantial cost savings for health insurance, especially if we assumed prescriptions shifted from escitalopram to generic citalopram. Finally, as evergreened medications are typically launched to the market before the patent of the original product expires - in the expectation of generic competition - it might be suitable to base the new product costs on the estimated price of the generic form, rather than on the current price of the originator. Moreover, health technology assessment agencies could redefine product innovation to reflect actual added benefits to patients and society. This might effectively make the evergreened product less cost effective and might help discourage this practice [42].

## Abbreviations

CI: confidence interval; DDD: defined daily dose; EGB: Echantillon généraliste de bénéficiaires; MADRS: Montgomery-Åsberg depression rating scale; NNT: number needed to treat; OR: odds ratio; RCTs: randomized controlled trials; SNIIR-AM: French National Health Insurance Inter-regime Information System

## Competing interests

The authors declare that they have no competing interests.

## Authors' contributions

AA contributed to the study design and performed the literature search, data collection and data interpretation. AA was also involved in drafting the manuscript. LT substantially contributed to the design, data collection and data analysis, and was also involved in the manuscript drafting and revising its intellectual content. GB contributed in gathering reimbursement data and its analysis, and also critically revised the manuscript for intellectual content. MD contributed to the conception and design of the study, and also was involved in the manuscript revision and approving its intellectual content. PR contributed substantially to the conception, design of the evergreening study and the interpretation of data, and was involved in revising the manuscript critically for intellectual content and has given the final approval of the version to be published. All authors have read and approved the manuscript for publication.

## Pre-publication history

The pre-publication history for this paper can be accessed here:

http://www.biomedcentral.com/1741-7015/10/142/prepub

## Supplementary Material

Additional file 1 **Section 1: Selection criteria and search strategy**. **Section 2**: Methods and analysis details. **Section 3**: Selected trials. **Section 4**: Network analysis of trials for direct and indirect comparison and the number of trials in each comparison. **Section 5**: Characteristics of trials. **Section 6**: Characteristics of trials across different comparisons. **Section 7**: Consumption and cost analyses for the citalopram, generic citalopram, escitalopram from the French national health insurance information system. **Section 8**: Meta-analysis for efficacy data of head-to-head trials. **Section 9**: Funnel plot for efficacy data for head-to-head trials. **Section 10**: Meta-analysis for acceptability data for head-to-head trials. **Section 11**: Meta-analysis for efficacy data for placebo-controlled trials. **Section 12**: Funnel plot for efficacy data for placebo-controlled trials. **Section 13**: Meta-analysis for acceptability data for placebo-controlled trials. **Section 14**: Consumption levels (monthly no. of prescriptions) between 2003 and 2011 in France. **Section 15**: Consumption levels (monthly defined daily dose (DDD) units) between 2003 and 2011 in France. **Section 16**: Consumption levels for escitalopram versus citalopram and its generic forms combined. **Section 17**: Total consumption of escitalopram, citalopram and its generic forms. Click here for file
