# Supplementary material for: Impact of evergreening on patients and health insurance: a meta analysis and reimbursement cost analysis of citalopram/escitalopram antidepressants
Source: BMC Med. 2012 Nov 20;10:142. doi: 10.1186/1741-7015-10-142 (PMC3520785; doi:10.1186/1741-7015-10-142)
Supplement: Additional file 1 — Section 1: Selection criteria and search strategy. Section 2: Methods and analysis details. Section 3: Selected trials. Section 4: Network analysis of trials for direct and indirect comparison and the number of trials in each comparison. Section 5: Characteristics of trials. Section 6: Characteristics of trials across different comparisons. Section 7: Consumption and cost analyses for the citalopram, generic citalopram, escitalopram from the French national health insurance information system. Section 8: Meta-analysis for efficacy data of head-to-head trials. Section 9: Funnel plot for efficacy data for head-to-head trials. Section 10: Meta-analysis for acceptability data for head-to-head trials. Section 11: Meta-analysis for efficacy data for placebo-controlled trials. Section 12: Funnel plot for efficacy data for placebo-controlled trials. Section 13: Meta-analysis for acceptability data for placebo-controlled trials. Section 14: Consumption levels (monthly no. of prescriptions) between 2003 and 2011 in France. Section 15: Consumption levels (monthly defined daily dose (DDD) units) between 2003 and 2011 in France. Section 16: Consumption levels for escitalopram versus citalopram and its generic forms combined. Section 17: Total consumption of escitalopram, citalopram and its generic forms. [file 1741-7015-10-142-S1.DOCX]

# Additional files

## **Section 1: selection criteria and search strategy**

**Identification and selection of reviews.** Eligible reviews assessed the efficacy of citalopram or escitalopram (any dosage) in adults (≥ 18 years old) with major depression via randomized controlled trials. Reviews that did not report efficacy assessment compared combination therapy or evaluated specific populations (eg, patients with concomitant chronic medical condition) and those in languages other than English were excluded.

First, we searched 4 electronic bibliographical databases (Cochrane Database of Systematic Reviews, Database of Abstracts of Reviews of Effects, MEDLINE via PubMed and EMBASE) for reviews published between January 2000 and March 2011. Search equations reflected Participants (major depression and synonyms) and Intervention (citalopram, escitalopram and their brand names) and included a filter to identify reviews in MEDLINE and EMBASE. The search strategies are reported below.

Second, we searched repositories of 4 national health technology agencies (UK National Institute for Health and Clinical Excellence, US Agency for Healthcare Research and Quality and the German Institute for Quality and Efficiency in Health Care). We also searched the repositories of the US Food and Drug Administration (Drugs@FDA database) and the French National Authority for Health (Haute Autorité de la Santé, Commission de la Transparence).

**Identification and selection of randomized controlled trials.** Eligible trials were randomized trials assessing the short-term efficacy (8-weeks) of escitalopram versus citalopram or escitalopram and/or citalopram versus placebo in patients with major depression. Fixed- and flexible dosage trials were eligible.

First, we screened reviews selected above and listed all included trials. Two reviewers read the corresponding report titles, abstracts and full texts, independently and in duplicate, to assess eligibility. In cases of disagreement at any step, consensus was reached by discussion.

Second, we searched for trial results published from March 2011 to February 2012 in MEDLINE via PubMed and EMBASE. Search equations reflected Participants (major depression and synonyms) and Intervention (citalopram, escitalopram and their brand names) and included the Cochrane Highly Sensitive Search filter for identifying randomized trials. The search strategies are reported below.

Finally, we searched for trial results in databases from the pharmaceutical companies commercializing citalopram and escitalopram (www.lundbecktrials.com, www.forestclinicaltrials.com). We also contacted Lundbeck France for a listing of clinical trials for the 2 medications.

**Search equation for MEDLINE**

| Participants | #1 ("Depressive Disorder"[Mesh] OR "Depressive Disorder, Major "[Mesh] OR "major depression"[Text Word] OR "major depressive disorder"[Text Word]) |
| --- | --- |
| Intervention | #2 ("Citalopram"[Mesh] OR "citalopram"[Text Word] OR "desmethylcitalopram"[Text Word] OR "seropram"[Text Word] OR "seroplex"[Text Word] OR "cipramil"[Text Word] OR "celexa"[Text Word] OR "Lexapro"[Text Word] OR "cipralex"[Text Word]) |
| Type of study | #3 (“systematic”[sb])  #4 Cochrane Highly Sensitive Search Strategy for identifying randomized trials in MEDLINE |
| Search equation for reviews | #1 AND #2 AND #3 |
| Search equation for trials | #1 AND#2 AND #4 |

**Search equation for EMBASE**

| Participants | #1 'major depression'/exp OR 'major depression' OR 'major depressive' |
| --- | --- |
| Intervention | #2 'citalopram'/exp OR 'escitalopram'/exp OR citalopram OR escitalopram OR seropram OR seroplex OR cipramil OR celexa OR lexapro OR cipralex OR lexamil OR lexam |
| Type of study | #3 'meta-analysis':ti OR 'meta-analysis':ab OR 'meta-analysis':de OR 'search':ti OR 'search':ab OR 'review':pt |
| Search equation | #1 AND #2 AND #3 |

**Search equation for Cochrane and DARE**

"major depression" OR "major depressive" OR "citalopram" OR " seropram" OR "seroplex" OR "escitalopram" in Title, Abstract, Keywords

## **Section 2: Methods and analysis details**

**Data extraction**: For each trial report, we extracted the publication status, publication year, the compared drugs, outcome assessment time, evaluated dosages (fixed or flexible), number of randomized patients, treatment responders, means and standard deviations for depression score at baseline and follow-up, change in depression score from baseline to follow-up, and age (mean, range) and sex proportion of subjects. This has been done by two independent reviewers; differences have been resolved by discussion.

**Outcome measures**: We assessed short-term treatment efficacy, that is, at the end of 8-week treatment. If 8-week assessment was not reported, we extracted outcome data for the closest time point reported. We extracted outcome data for the Montgomery-Åsberg depression rating scale (MADRS) and the Hamilton scale. When reports described results from both rating scales, we used the MADRS results. Efficacy was assessed by the proportion of responders in each treatment group, defined as patients with a decrease in depression score from baseline to follow-up of at least 50%. The numerator was the number of responders among the “efficacy” subset (ie, patients who received at least one dose of a drug and had at least one follow-up visit) and, when used, derived by the Last Observation Carried Forward (LOCF). The denominator was the number of randomly assigned participants. Subjects not included in the efficacy subset and drop-outs (when LOCF was not used) were assumed to be non-responders. In the absence of binary outcome data, we calculated the proportion of responders according to validated imputation methods. We computed the NNT from the combined ORs and by considering low and high response rates for the control group, defined as the lower and upper bounds of the 95% CI for the combined response rate across control groups in the meta-analysis.

We assessed treatment acceptability by the proportion of patients who did not drop out of the allocated treatment during the short-term treatment period (completers).

## **Section 3: Selected trials.**

| **Trial ID** | **Reference** | **Publication Status** |
| --- | --- | --- |
| **Citalopram-placebo** | | |
| 29060/785 | [1] | Unpublished |
| 89306 | [2] | Unpublished |
| 86141, Nyth 1992 | [3, 4] | Unpublished /Published |
| 85A, Mendels 1999 | [5, 6] | Unpublished /Published |
| 91206, Feighner 1999 | [7, 8] | Unpublished /Published |
| 89303, Montgomery 1992 | [9, 10] | Unpublished /Published |
| CIT-MD-03, Roose 2004 | [11, 12] | Unpublished /Published |
| **Escitalopram-placebo** | | |
| AK130927, Clayton 2005 | [13, 14] | Unpublished /Published |
| AK130926, Clayton 2005 | [13, 14] | Unpublished /Published |
| 99001, Wade 2002 | [15, 16] | Unpublished /Published |
| 99024,Kasper 2005 | [17, 18] | Unpublished /Published |
| Bose 2008 | [19] | Published |
| F1J-US-HMCR,Pigott 2007, Nierenberg 2007 | [20-22] | Unpublished /Published |
| SCT-MD-26 | [23] | Unpublished |
| SCT-MD-35 | [24] | Unpublished |
| SCT-MD-27 | [25] | Unpublished |
| **Escitalopram -citalopram** | | |
| 99022, Colonna 2005 | [26, 27] | Unpublished /Published |
| Moore 2005 | [28] | Published |
| Ou 2011 | [29] | Published |
| Yevtushenko 2007 | [30] | Published |
| **Escitalopram-citalopram-placebo** | | |
| 99003, Montgomery 2001, Lepola 2003 | [31-33] | Unpublished/Published |
| SCT-MD-02 | [34] | Unpublished |
| SCT-MD-01, Burke 2002 | [35, 36] | Unpublished/Published |

**References**

All provided links were accessed on April 11, 2012.

1. GSK Pharmaceuticals: **29060/785**. In: *Clinical trials registry.* GSK 2001: <http://www.gsk-clinicalstudyregister.com/result_detail.jsp;jsessionid=4EDCA4808236FF4808292B4808236B4808231B4429822C4808239A4808233?protocolId=4829060%4808232F4808785&studyId=5898670D-4808237AD4808235-4808240E4808236-B4808802-4808726A4808232A4808273CABD&compound=paroxetine>

2. FDA: **89306**. In: *Statistical and Medical evaluation.* 2001: <http://www.accessdata.fda.gov/drugsatfda_docs/nda/98/020822a_medr_P020822.pdf>.

3. Nyth AL, Gottfries CG, Lyby K, Smedegaard-Andersen L, Gylding-Sabroe J, Kristensen M, Refsum HE, Ofsti E, Eriksson S, Syversen S: **A controlled multicenter clinical study of citalopram and placebo in elderly depressed patients with and without concomitant dementia**. *Acta Psychiatr Scand* 1992, **86**(2):138-145.

4. FDA: **86141** In: *Medical evaluation*

2001: <http://www.accessdata.fda.gov/drugsatfda_docs/nda/98/020822a_medr_P020821.pdf>

5. Mendels J, Kiev A, Fabre LF: **Double-blind comparison of citalopram and placebo in depressed outpatients with melancholia**. *Depress Anxiety* 1999, **9**(2):54-60.

6. FDA: **85A**. In: *Statistical evaluation.* 2001: <http://www.accessdata.fda.gov/drugsatfda_docs/nda/98/020822a_statr_P020823.pdf>

7. Feighner JP, Overo K: **Multicenter, placebo-controlled, fixed-dose study of citalopram in moderate-to-severe depression**. *J Clin Psychiatry* 1999, **60**(12):824-830.

8. FDA: **91206**. In: *Statistical Evaluation.* 2001: <http://www.accessdata.fda.gov/drugsatfda_docs/nda/98/020822a_medr_P020822.pdf>

9. Montgomery SA, Rasmussen JG, Lyby K, Connor P, Tanghoj P: **Dose response relationship of citalopram 20 mg, citalopram 40 mg and placebo in the treatment of moderate and severe depression**. *Int Clin Psychopharmacol* 1992, **6 Suppl 5**:65-70.

10. FDA: **89303**. In: *Statistical evaluation.* FDA; 1992: <http://www.accessdata.fda.gov/drugsatfda_docs/nda/98/020822a_medr_P020822.pdf?bcsi_scan_CBA020824F020892DB020823F020863E020822=020820&bcsi_scan_filename=020822a_medr_P020822.pdf>

11. Roose SP, Sackeim HA, Krishnan KR, Pollock BG, Alexopoulos G, Lavretsky H, Katz IR, Hakkarainen H: **Antidepressant pharmacotherapy in the treatment of depression in the very old: a randomized, placebo-controlled trial**. *Am J Psychiatry* 2004, **161**(11):2050-2059.

12. Forest Laboratories: **CIT-MD-03**. In: *Clinical Trial Registry.* <http://www.forestclinicaltrials.com/CTR/CTRController/CTRViewPdf?_file_id=scsr/SCSR_CIT-MD-03_final.pdf>

13. Clayton AH, Croft HA, Horrigan JP, Wightman DS, Krishen A, Richard NE, Modell JG: **Bupropion extended release compared with escitalopram: effects on sexual functioning and antidepressant efficacy in 2 randomized, double-blind, placebo-controlled studies**. *J Clin Psychiatry* 2006, **67**(5):736-746.

14. GSK Pharmaceuticals: **WELL_AK130926**. In: *Clinical Trial Registry.* 2005: <http://www.google.fr/url?sa=t&rct=j&q=&esrc=s&source=web&cd=1&cts=1331652480045&sqi=1331652480042&ved=1331652480040CCcQFjAA&url=http%1331652480043A%1331652480042F%1331652480042Fdownload.gsk-clinicalstudyregister.com%1331652480042Ffiles%1331652480042F1331652420466.pdf&ei=ymZfT1331652480044jmCILrOZ-WiOYH&usg=AFQjCNE1331652480048VD-Qj1331652480045h_J-vTUoG1331652480042OpTrlpy1331652480042Qg&sig1331652480042=-Hds1331652480040_HmXy1331652480043NpPN1331652480047ydqfvg>.

15. Wade A, Michael Lemming O, Bang Hedegaard K: **Escitalopram 10 mg/day is effective and well tolerated in a placebo-controlled study in depression in primary care**. *Int Clin Psychopharmacol* 2002, **17**(3):95-102.

16. FDA: **99001**. In: *Statistical Evaluation.* FDA; 2000: <http://www.accessdata.fda.gov/drugsatfda_docs/nda/2003/021323_S021007&021365_S021001_LEXAPRO_TABS.pdf>

17. Kasper S, de Swart H, Friis Andersen H: **Escitalopram in the treatment of depressed elderly patients**. *Am J Geriatr Psychiatry* 2005, **13**(10):884-891.

18. Lundbeck Pharmaceutical: **99024**. In: *Clinical Trial registry.* 2001: <http://www.lundbecktrials.com/Data/PDFs/99024_CTRS_final_99030Dec92005.pdf>

19. Bose A, Li D, Gandhi C: **Escitalopram in the acute treatment of depressed patients aged 60 years or older**. *Am J Geriatr Psychiatry* 2008, **16**(1):14-20.

20. Pigott TA, Prakash A, Arnold LM, Aaronson ST, Mallinckrodt CH, Wohlreich MM: **Duloxetine versus escitalopram and placebo: an 8-month, double-blind trial in patients with major depressive disorder**. *Curr Med Res Opin* 2007, **23**(6):1303-1318.

21. Nierenberg AA, Greist JH, Mallinckrodt CH, Prakash A, Sambunaris A, Tollefson GD, Wohlreich MM: **Duloxetine versus escitalopram and placebo in the treatment of patients with major depressive disorder: onset of antidepressant action, a non-inferiority study**. *Curr Med Res Opin* 2007, **23**(2):401-416.

22. lilly E: **F1J-US-HMCR**. In: *Clinical Trial Registry.* 2007.

23. Forest Laboratories: **SCT-MD-26**. In: *Clinical Trial Registry.* 2002: <http://www.forestclinicaltrials.com/CTR/CTRController/CTRViewPdf?_file_id=scsr/SCSR_SCT-MD-26_final.pdf>

24. Forest Laboratories: **SCT-MD-35**. In: *Clinical Trial Registry.* 2007: <http://www.forestclinicaltrials.com/CTR/CTRController/CTRViewPdf?_file_id=scsr/SCSR_SCT-MD-35_final.pdf>

25. Forest Laboratories: **SCT-MD-27**. In: *Clinical Trial Registry.* 2005: <http://www.forestclinicaltrials.com/CTR/CTRController/CTRViewPdf?_file_id=scsr/SCSR_SCT-MD-27_final.pdf>

26. Colonna L, Andersen HF, Reines EH: **A randomized, double-blind, 24-week study of escitalopram (10 mg/day) versus citalopram (20 mg/day) in primary care patients with major depressive disorder**. *Curr Med Res Opin* 2005, **21**(10):1659-1668.

27. Lundbeck Pharmaceutical: **99022**. In: *Clinical Trial registry.* Lundbeck Pharmaceutical; 2002: <http://www.lundbecktrials.com/Data/PDFs/99022_CTRS_final_99030Dec92005.pdf>

28. Moore N, Verdoux H, Fantino B: **Prospective, multicentre, randomized, double-blind study of the efficacy of escitalopram versus citalopram in outpatient treatment of major depressive disorder**. *Int Clin Psychopharmacol* 2005, **20**(3):131-137.

29. Ou JJ, Xun GL, Wu RR, Li LH, Fang MS, Zhang HG, Xie SP, Shi JG, Du B, Yuan XQ *et al*: **Efficacy and safety of escitalopram versus citalopram in major depressive disorder: a 6-week, multicenter, randomized, double-blind, flexible-dose study**. *Psychopharmacology (Berl)* 2011, **213**(2-3):639-646.

30. Yevtushenko VY, Belous AI, Yevtushenko YG, Gusinin SE, Buzik OJ, Agibalova TV: **Efficacy and tolerability of escitalopram versus citalopram in major depressive disorder: a 6-week, multicenter, prospective, randomized, double-blind, active-controlled study in adult outpatients**. *Clin Ther* 2007, **29**(11):2319-2332.

31. Montgomery SA, Loft H, Sanchez C, Reines EH, Papp M: **Escitalopram (S-enantiomer of citalopram): clinical efficacy and onset of action predicted from a rat model**. *Pharmacol Toxicol* 2001, **88**(5):282-286.

32. Lepola UM, Loft H, Reines EH: **Escitalopram (10-20 mg/day) is effective and well tolerated in a placebo-controlled study in depression in primary care**. *Int Clin Psychopharmacol* 2003, **18**(4):211-217.

33. Center of Drug Evaluation and Research: **99003**. In: *Statistical and medical evaluation.* FDA; 2001: <http://www.accessdata.fda.gov/drugsatfda_docs/nda/2002/2021-2323.pdf_Lexapro_Medr_P2001.pdf>.

34. Forest Laboratories: **SCT-MD-02**. In: *Clinical Trial Registry.* 2005: <http://www.forestclinicaltrials.com/CTR/CTRController/CTRViewPdf?_file_id=scsr/SCSR_SCT-MD-02_final.pdf>

35. Burke WJ, Gergel I, Bose A: **Fixed-dose trial of the single isomer SSRI escitalopram in depressed outpatients**. *J Clin Psychiatry* 2002, **63**(4):331-336.

36. Center of Drug Evaluation and Research: **SCT-MD-01**. In: *Statistical evaluation.* FDA; 2002: <http://www.accessdata.fda.gov/drugsatfda_docs/nda/2002/2021-2323.pdf_Lexapro_Statr.pdf>.

**Section 4: Network analysis of trials for direct and indirect comparison and the number of trials in each comparison.
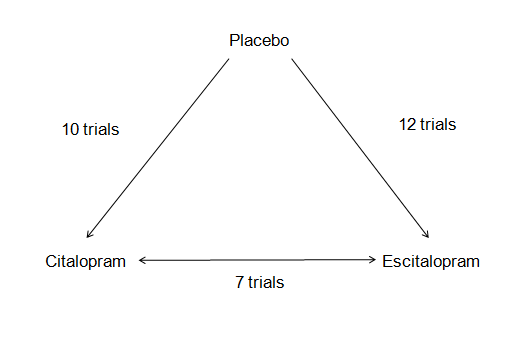
**

## **Section 5: Characteristics of trials.**

| Trial ID | Age, mean (yr) | Male, % | Scale used in the analysis | Baseline  depression severity, mean | Setting | Elderly  specific  population | Dosage | Outcome  measurement  delay (weeks) | Funder |
| --- | --- | --- | --- | --- | --- | --- | --- | --- | --- |
| **Citalopram-placebo** | | | | | | | | | |
| 89306 | 42.7 | 32 | MADRS | 32.1 | In- and outpatients | No | Fixed | 6 | Forest Lab |
| 29060/785 | 39.4 | 43 | MADRS | 31.4 | NC | No | Fixed | 6 | GSK |
| 85A, Mendles 1999 | 43 | 66 | HAMD | 33.7 | Outpatients | No | Flexible | 4 | Pfizer |
| 86141, Nyth 1992 | 77.2 | 29 | MADRS | 25.2 | In- and outpatients | Yes | Flexible | 6 | Lundbeck |
| 89303, Montgomery 1992 | 43.2 | 30 | MADRS | 24.7 | In- and outpatients | No | Fixed | 6 | Lundbeck |
| 91206, Feighner 1999 | 39.5 | 40 | MADRS | 27.3 | Outpatients | No | Fixed | 6 | Lundbeck |
| CIT-MD-03, Roose 2004 | 79.5 | 42 | MADRS | 24.7 | NC | Yes | Flexible | 8 | Forest Lab |
| **Escitalopram-placebo** | | | | | | | | | |
| AK130926, Clayton 2005 | 36.5 | 35 | HAMD | 24.1 | Outpatients | No | Flexible | 8 | GSK |
| AK130927, Clayton 2005 | 35 | 38 | HAMD | 24.1 | Outpatients | No | Flexible | 8 | GSK |
| 99001, Wade 2002 | 40.5 | 24 | MADRS | 28.9 | Outpatients | No | Fixed | 8 | Forest lab |
| 99024, Kasper 2005 | 75 | 24 | MADRS | 28.4 | In- and outpatients | Yes | Fixed | 8 | Lundbeck |
| Bose 2008 | 68.3 | 40 | MADRS | 28.9 | NC | Yes | Flexible | 12 | Forest Lab |
| F1J-US-HMCR, Pigott 2007, Nieremberg 2007 | 42.9 | 33 | HAMD | 17.8 | Outpatients | No | Flexible | 8 | Eli Lilly |
| SCT-MD-26 | 38.4 | 37 | MADRS | 30.4 | NC | No | Flexible | 8 | Forest Lab |
| SCT-MD-27 | 40 | 47 | MADRS | 30.6 | NC | No | Flexible | 8 | Forest Lab |
| SCT-MD-35 | 41.4 | 45 | MADRS | 30.1 | Outpatients | No | Fixed | 8 | Forest Lab |
| **Escitalopram-Citalopram** | | | | | | | | | |
| 99022, Colonna 2005 | 46 | 25 | MADRS | 29.9 | Outpatients | No | Fixed | 8 | Lundbeck |
| Moore 2005 | 45.1 | 33 | MADRS | 36 | Outpatients | No | Fixed | 8 | Lundbeck |
| Ou 2011 | 36.5 | 43 | HAMD | 23.2 | In- and outpatients | No | Flexible | 6 | Chinese National Institute of Pharmaceutical Research and Development |
| Yevtushenko 2007 | 34.9 | 41 | MADRS | 35.2 | Outpatients | No | Fixed | 6 | Arbacom |
| **Escitalopram-Citalopram-Placebo** | | | | | | | | | |
| 99003, Montgomery 2001, Lepola 2003 | 43.3 | 27 | MADRS | 28.9 | Outpatients | No | Flexible | 8 | Forest Lab |
| SCT-MD-01, Burke 2002 | 40.1 | 34 | MADRS | 28.6 | Outpatients | No | Fixed | 8 | Forest Lab |
| SCT-MD-02 | 41.9 | 48 | MADRS | 28.6 | Outpatients | No | Flexible | 8 | Forest Lab |

MADRS= [Montgomery–Åsberg Depression Rating Scale](http://www.google.fr/url?sa=t&rct=j&q=&esrc=s&source=web&cd=1&ved=0CC0QFjAA&url=http%3A%2F%2Fen.wikipedia.org%2Fwiki%2FMontgomery%25E2%2580%2593%25C3%2585sberg_Depression_Rating_Scale&ei=AFx5T6KdDMmP0AWk2rizDQ&usg=AFQjCNFqvAT3Tkuw9x_buHGxNIkBXPppig&sig2=NQx_RkEnPGJ02e3XUMvPHQ)

HAMD= Hamilton Rating Scale for Depression

NC= unclear

## **Section 6: Characteristics of trials by different comparisons.**

|  | Age, mean (yrs) | Male, % | Baseline depression severity (MADRS)*, mean | Fixed dosage regimen | 8-week outcome assessment |
| --- | --- | --- | --- | --- | --- |
| Citalopram vs placebo (10 trials) | 45.6 | 40% | 28.9 | 5 trials (50%) | 4 trials (40%) |
| Escitalopram vs placebo (12 trials) | 45.9 | 36% | 29.2 | 4 trials (33%) | 11 trials (91.6%) |
| Escitalopram vs citalopram (7 trials) | 41.2 | 34% | 31.2 | 4 trials (57%) | 5 trials (71%) |

## *MADRS, Montgomery-Åsberg depression rating scale

## **Section 7: Consumption and cost analyses for the citalopram, its generic forms, and escitalopram from the French national health insurance information system.**

|  | Number of reimbursements | | | DDD* units  (in thousands) | | | Annual reimbursement cost  (in millions of euros)† | | |
| --- | --- | --- | --- | --- | --- | --- | --- | --- | --- |
| Year | Citalopram | Citalopram generic drugs | Escitalopram | Citalopram | Citalopram generic drugs | Escitalopram | Citalopram | Citalopram generic drugs | Escitalopram |
| 2003 | 2,634,520 | 9,215 | – | 89,166 | 342 | – | 60.9 | 0.1 | – |
| 2004 | 2,184,828 | 87,2903 | – | 73,905 | 30,348 | – | 50.8 | 14.1 | – |
| 2005 | 1,335,787 | 160,4962 | 457,355 | 45,126 | 55,210 | 15,732 | 31.5 | 25.6 | 8.6 |
| 2006 | 757,861 | 1,676,548 | 1,709,140 | 25,200 | 57,179 | 62,095 | 16.0 | 24.4 | 33.4 |
| 2007 | 431,068 | 1,813,318 | 2,626,081 | 14,004 | 61,264 | 98,888 | 8.6 | 24.6 | 50.7 |
| 2008 | 263,646 | 1,851,633 | 3,460,475 | 8,705 | 61,769 | 125,788 | 5.3 | 23.0 | 63.0 |
| 2009 | 247,447 | 1,735,815 | 4,273,723 | 7,801 | 57,533 | 149,165 | 4.6 | 20.2 | 75.4 |
| 2010 | 237,262 | 1,739,404 | 5,486,320 | 7,607 | 58,567 | 193,839 | 4.4 | 20.3 | 96.8 |

*DDD, defined daily dosage: For a given reimbursement, the corresponding number of DDD units was the number of pills × the dosage × the number of boxes reimbursed divided by the DDD. For instance, the reimbursement of 2 boxes of 28 pills of escitalopram, 20 mg, would be 112 DDD units (28×20×2/10).

† For a given reimbursement, the corresponding cost was the number of boxes reimbursed × the direct unit price × the amount of reimbursement coverage.

## **Section 8: Meta-analysis of efficacy data for head-to-head trials.**

Odds ratio > 1 favours escitalopram over citalopram

- I-V : [*Inverse*-variance fixed effect model.](https://www.google.fr/search?hl=en&safe=off&client=firefox-a&hs=Ngo&rls=org.mozilla:en-US:official&sa=X&ei=i1XoT427JM_zsgaynPCkAQ&ved=0CBEQBSgA&q=-%09I-V+%3A+Inverse-variance+fixed+effect+model+-%09D%2BL%3A+random+effect+dersimonian&spell=1)
- D+L: random effect dersimonian.
- For the efficacy analysis: The 95% prediction interval was 0.39–6.62, which overlapped 1 and so in some settings, escitalopram may not have been superior to citalopram.
- Heterogeneity was considerable across trials (I²=80%; τ²=0.26), but mainly because of one trial, Yevtushenko 2007, which showed outlying results. After excluding this trial, the heterogeneity was moderate (I²=39% and τ²=0.04) and the meta-analysis showed significant superiority of escitalopram over citalopram (random-effects OR 1.30 [1.02–1.66]).

## **Section 9: Funnel plot of efficacy data for head-to-head trials.**

- Criteria to apply asymmetry tests were not met, because of fewer than 10 trials, the considerable heterogeneity and an insufficiently large difference in precision of the largest and smallest study.
- The funnel plot of the 7 comparisons did not reveal asymmetry, although Yevtushenko 2007 had the largest standard error and showed the largest outlying treatment effect.

## **Section 10: Meta-analysis of acceptability data for head-to-head trials**

##

Odds ratio > 1 favours escitalopram over citalopram

## **Section 11: Meta-analysis of efficacy data for placebo-controlled trials.**

Odds ratio > 1 favours escitalopram or citalopram over placebo

## **Section 12: Funnel plot for efficacy data for placebo-controlled trials.**

## **Section 13: Meta-analysis for acceptability data for placebo-controlled trials.**

Odds ratio > 1 favours escitalopram or citalopram over placebo

## **Section 14: Consumption levels (monthly no. of prescriptions) between 2003 and 2011 in France.**

##

## **Section 15: Consumption levels (monthly defined daily dosage [DDD] units) between 2003 and 2011 in France.**

## **Section 16: Monthly consumption levels for escitalopram versus citalopram and its generic forms combined.**

##

## **Section 17: Total monthly consumption of escitalopram, citalopram and its generic forms combined.**
